# Perceptions of the national school holiday programme (Holiday Activities and Food) in England as experienced by local government, service providers and users of the service: a mixed-methods study

**DOI:** 10.1186/s12889-026-27699-1

**Published:** 2026-05-30

**Authors:** Lorna Hatch, Laura Tinner, Cecilia Khofi-Szeremley, Florence Darling, Sophie Clohessy, Jessica Tanner, Marie Murphy, Miranda Pallan, Carolyn Summerbell, Laura Mazzoli-Smith, Irina Pokhilenko, Russell Jago

**Affiliations:** 1 Population Health Sciences, Bristol Medical School, University of Bristol, Canynge Hall, Bristol, BS8 2PN UK; 2 Department of Sport and Exercise Sciences, Durham University, Durham, UK; 3 Centre for Translational Research in Public Health, Newcastle upon Tyne, UK; 4 Department of Applied Health Sciences, University of Birmingham, Birmingham, UK; 5 School of Education, Durham University, Durham, UK; 6 Centre for Economics of Obesity, University of Birmingham, Birmingham, UK; 7 The National Institute for Health and Care Research, Applied Research Collaboration West (NIHR ARC West), University Hospitals Bristol and Weston NHS Foundation Trust, Bristol, UK

**Keywords:** Policy, Underserved, Low income, Children, Food insecurity, Public health

## Abstract

**Background:**

School holidays can be a period of isolation and difficulty for children from low-income families. Limited opportunities for enrichment activities, and food insecurity, mean that children’s health and well-being can suffer. There is also potential learning loss, particularly during longer holiday periods. The Holiday Activities & Food (HAF) programme is a government-funded (> £200 M/y) initiative in England which aims to provide healthy meals and activities to free school meal (FSM)-eligible children during school holidays. Existing evaluations of the programme have identified varied provision across England, with challenges such as quality of delivery, engagement and attendance. Evidence is needed to understand how the programme could be improved, from the perspectives of those involved in delivery and families who attend. In this study we present data which addresses this gap and can inform relevant policy and practice.

**Method:**

We purposively sampled participants from across the Southwest, Midlands and Northeast of England. Interviews were conducted with local government Political Leads (*n*=5), HAF programme Leads (*n*=11), HAF club Providers (*n*=12), parents (*n*=10), and children with Special Educational Needs and Disabilities (SEND; 9-12y; *n*=2). Primary school children (8-12y; *n*=15) participated in focus groups. Data were analysed using the Framework Method. HAF Leads (*n*=9) completed an economic survey.

**Results:**

Five key themes were identified: (1) Eligibility, reach and attendance: Open-access provision is needed to reduce stigma and improve attendance. (2) The importance of relationships: Families and communities need to work together; (3) Delivering a high-quality, inclusive HAF programme: there is a need for flexible choice-led provision; (4) Schools as trusted advocates: Schools can support reach and recruitment of the HAF programme; and (5) Evaluation of HAF: Current evaluations are insufficient and a comprehensive evaluation with children, families and communities is needed.

**Conclusion:**

To ensure that families are receiving the support they need, we recommend that changes to the programme allow community organisations to have agency to decide how best to work with their families, achieved by greater flexibility within the national guidelines and criteria. Moreover, we recommend *poverty-proofed*, place-based holiday provision that is iteratively developed with the involvement of local families.

**Supplementary Information:**

The online version contains supplementary material available at 10.1186/s12889-026-27699-1.

## Background

Currently, 4.3 million children in the UK are growing up in poverty [1] and inequalities in health and education are widening. The school holidays are a critical time for children as they can bring increased pressure on families due to extra costs for meals, as well as a lack of affordable and accessible childcare provision, and affordable places for children to play and engage in social and physical activities [2, 3]. There is also evidence of widening inequalities and learning loss during school closure periods [4–6]. Therefore, during school holidays disadvantaged children are at further risk of experiencing hunger, poor diet, social isolation, a limited learning experience, and sedentary lifestyles [7], further increasing inequalities and exacerbating food insecurity [8]. These negative experiences impact the growth, development, well-being and educational attainment of children [9–13].

In an attempt to reduce inequalities in school holiday experiences, the UK government introduced the Holiday Activities and Food (HAF) programme in England, to support children from low-income families in receipt of benefits-related free-school meals (FSM) [14]. Following successful pilots, in 2021 the Government announced a 3-year funding settlement of > £200 M each year across all 152 upper tier local authorities (LAs) in England to deliver holiday clubs in the spring, summer and winter holidays. Funding is allocated based on the proportion of children eligible for FSM and each LA is responsible for appointing a HAF Lead who coordinates all elements of the programme including mapping provision, commissioning providers and data collection [15]. Each year since its conception, the Department for Education have reviewed and published the aims, ‘core offer’ and ‘standards for holiday provision’ on the HAF programme website [16]. The 2024 aims of the HAF programme are to: provide children with a healthy and nutritious meal (which meets School Food Standards [17]); increase their physical activity levels and awareness of nutrition; promote a healthy lifestyle and positive behaviours; provide safe and secure environments and support; offer enrichment activities that support their development; and ensure that children are happy, socialising and ready to return to school [18].

Previous research has shown that the HAF programme holds great potential to reduce children’s experiences of food insecurity and social isolation [19] as well as offer wider support and benefit to families [12, 15, 20, 21]. Evidence suggests, however, that there is variation in the quality of provision across LAs, as well as challenges to the successful delivery of the programme, including time and funding constraints, restrictive eligibility criteria, low attendance rates and failing to provide a ‘healthy and nutritious’ meal [22, 23]. Additionally, HAF is an evolving programme situated within a complex system and wider political context, with increasing expectations around the standards of provision from the government over time [18].

There is a need for greater understanding of the perspectives of those responsible for delivering the HAF programme and eligible families across England including what is working well and less well, what is most valuable and impactful, and how challenges can be overcome. Additionally, we need to understand how we can ensure the programme is inclusive and accessible to all eligible children.

The aims of this project were to understand the perceptions of the HAF programme as experienced by those involved in planning and delivering the programme as well as those attending the programme. We intend to inform further in-depth work in this area examining challenges to the programme, and consequently the co-development of guidance and recommendations for a successful and sustainable, high-quality HAF programme.

## Methods

### Study design

A qualitative interview and focus group study in England and an economics survey of selected interview participants to explore financial data on HAF programme delivery.

### Sampling and recruitment

We used purposive sampling to capture diversity of experience across the study sites within Northeast, Midlands, and Southwest England, aiming also to include participants from city/urban, rural and coastal areas, which we achieved. Participants came from two different stakeholder groups involved in the HAF programme: we firstly recruited stakeholders involved in delivering HAF (see additional file 1 for definitions of the roles), this included HAF Leads (*n* = 11), Political Leads (*n* = 5), and HAF Providers (holiday club staff; *n* = 12), representing 12 local authority areas across the three geographic regions. Subsequently, we recruited families engaging in HAF provision: children aged 4–12 (*n* = 17) and parents (*n* = 10). For both participant groups, we aimed for a diverse sample with regards to location, gender, age, and ethnicity. This was somewhat achieved (see Table 1), though for the staff delivery stakeholders the sample was mostly female, aged 40–49 and White British. Participants were recruited through existing contacts, online searches, as well as HAF Lead and Provider participants who recommended Political Leads and family attendees. We selected families and HAF Providers from a range of HAF clubs to capture a broad representation of perspectives and experiences. This included variations in location from rural, coastal and urban contexts, size, activity type and setting (additional file 2).

**Table 1:** Participant demographics

Participant type	Region*		Gender		Age range
LA Political Leads		*n* =		*n* =	
	NE	2	Female	5	50–71 y
	M	2	Male	0	-
	SW	1			
HAF Leads					
	NE	3	Female	7	32–52 y
	M	5	Male	4	37–63 y
	SW	3			
HAF Providers					
	NE	4	Female	7	28–57 y
	M	3	Male	4	28–54 y
	SW	5	Undisclosed	1	43 y
Parents					
	NE	3	Female	7	34–48 y
	M	5	Male	3	27–43 y
	SW	2			
Children					
	NE	8	Female	6	8–11 y
	M	7	Male	11	9–12 y
	SW	2			

*NE North East, *M *Midlands, *SW *South West

### Data collection

Data were collected between October 2023 and May 2024. Interviews were conducted to explore the perceptions of the HAF programme by HAF Leads, LA Political Leads, HAF Providers and parents. We also conducted interviews with two children with SEND. All interviews were conducted online apart from one with a child with SEND which was conducted in person, based on their desire to participate but feeling less comfortable in a focus group setting or online. Interviews ranged between 30 and 60 min, with the majority being around 45 min. Focus groups were used to explore the perceptions of the HAF programme of children without SEND. Two of the three focus groups took place in person at HAF club sites. The other took place online. Decisions around format were informed by participant preference (i.e., all adults preferred online interviews) and our prior knowledge that focus groups with children would be more engaging in person, although there were no notable differences in the data collected at the online and in person focus groups. The focus groups involved 4–6 children and lasted 37–50 min. A semi-structured topic guide, with open-ended questions and prompts, was developed for each participant group which allowed an understanding of their individual perceptions and experiences of the programme (see additional files 3–8).

An economic survey was developed by the study team to supplement the interview data and understand the nature of HAF programme delivery across the country, including how the programme is financed, and what costs are associated with its delivery across the participating LAs. The survey specifically focused on HAF provision during the summer 2023, as data were being collected in Autumn 2023 and the programme is delivered across all LAs during this school holiday period. The study health economist (IP) developed the initial version of the survey, which was reviewed by the study team. Surveys were sent to all eleven HAF leads who participated in an interview but only 9 returned representing data from 9 different LA areas with 8 being completed by LA-based HAF lead and 1 completed by external HAF lead.

### Analysis

Inductive and deductive thematic analysis [24] was conducted and the Framework Method [25] was used to organise, code and map the data. The Framework Method involved seven distinct stages (see additional file 9). NVivo version 12 software (QSR International) was used to aid data management.

The surveys were analysed by the team economist (IP) and findings are integrated narratively throughout where they contribute to our theme generation, with further findings summarised in additional file 11. The survey data was analysed and reported descriptively.

#### Positionality

Who the researcher is makes a difference to the interpretation of findings and it is crucial for reflexive practice to be transparent about any contextual intersecting factors between us and participants [26] The researchers who conducted the interviews had some familiarity with the HAF programme, with one having previously been involved in HAF club delivery and the others through attending HAF clubs in preparation for the interviews. Additional researchers were involved in data analysis, most of whom were unfamiliar with HAF prior to the project. In the context of the parent interviews and child focus groups, the positionality of the researchers as having not used the HAF clubs (and currently not experiencing poverty) created an ‘outsider’ status [27] that may have been useful for encouraging participants to go into greater detail, but ultimately limits the trustworthiness of our interpretations and illuminates inherent power imbalances in the researcher/participant relationship. We aimed to approach a level of trustworthiness in our arguments through reviewing preliminary findings with our advisory groups and revisiting our Framework if their insider knowledge indicated alternate interpretations.

## Results

### Participant characteristics

Table 1 displays our sample characteristics.

### The context of the HAF programme

HAF is delivered through LAs, meaning there is significant variation in delivery to fit with differing LA set up, local population characteristics, and different local needs and resources. However, local HAF teams, and their capacity, also differ between areas. For example, while some local authority areas have a HAF Lead and a team of support staff coordinating programme delivery, other areas have only a HAF Lead who is solely responsible for co-ordinating delivery. Moreover, there is diversity between areas in the extent of strategic input and support HAF Leads receive from their LA Political Lead. The majority of HAF Leads discussed the direction and support gained from a steering group of stakeholders, however, many of the participants mentioned challenges with balancing HAF and its set funding with wider LA policies and strategy. They emphasised a need for more strategic, joined-up working across national and local policy departments and portfolios to support these families through and beyond HAF: *“It just feels as if…are we putting loads of sticking plasters over something that actually… needs a bigger…a more strategic look at”* (Political Lead 5).

Financially, it has been, and continues to be, a difficult time for families. Many LAs have been working on anti-poverty strategies and some had local campaigns/holiday provision pre-HAF. Many consider HAF vital and valuable to support families, yet participants stressed insufficient funding relative to the need and costs, and crucially insufficient funds to reach the desired numbers of children and deliver the (ever-expanding) expected components of the programme (according to Department for Education guidelines). More broadly, this challenging financial landscape was highlighted as ‘political’ and thus resulted in HAF potentially trying to *“do too much”*, where higher level policies to tackle nationwide poverty would be better placed: “*‘Oh*,* well*,* don’t worry because we’ve given you this Holiday and Food programme’. Well*,* it doesn’t anywhere near address the needs of people out there.”* (Political Lead 3). Across the nine surveyed LAs, the mean grant received from DfE to deliver the HAF programme in the summer 2023 was £1,546,866, ranging between £200,000 - £5,000,000. Additional financial and/or in-kind support was provided by LAs/councils, housing associations, universities, food providers, local businesses, supermarkets, and third sector organisations. However, it was not possible to provide financial estimates of this additional support. Further details on the context of HAF that were derived from the survey can be found in additional file 10.

### Themes from data

#### Theme 1: eligibility, reach and attendance

The interconnected issues of eligibility, reach and attendance were highlighted as the greatest challenges related to HAF clubs. *Eligibility* refers to the criteria used to decide whether children can attend a HAF club; *reach* refers to the efforts of HAF delivery teams to ensure eligible families are aware and have the opportunity to access HAF; *attendance* covers challenges around whether children who are aware of HAF (have been ‘reached’) are attending a club.

##### Eligibility

Several Political Leads, HAF Leads and HAF Providers felt uncomfortable with the fact that to be eligible to attend a HAF club, children had to be eligible for free school meals (FSM). The UK government currently funds FSM for children in Key Stage 2 (7–11 years of age) if they are receiving certain benefits and are below a defined income threshold. HAF Leads highlighted the inadequacy of the current FSM threshold, arguing that many more children are living in low-income families and should be entitled to FSMs and to attend HAF provision: “*There’s a lot of kids*,* a lot of families out there that are on the brink”* (HAF Lead 8). Using FSM as the main criteria was seen as challenging because there are so many families living in financial insecurity, and children who would benefit from HAF, who are not eligible including: children with SEND, children in LA care and those at risk of school exclusion.

LAs have the discretion to use up to 15% of their HAF funding on children who are not in receipt of benefits-related FSM. However, HAF Leads felt that they needed more flexibility to offer more places and that the current guidance around 15% was too vague. HAF Leads and Providers also discussed the difficulty in enforcing the FSM policy at the grassroots level, which meant explaining to families and children, who they felt should be eligible based on their needs, why they couldn’t take part: “*That is a really hard thing to communicate with families*,* with everyone*,* because they have no understanding of what our 15% looks like*,* how could they?”* (HAF Lead 1). Five of the nine survey respondents indicated that they LA funded or subsidised attendance for ineligible children, including: children with Education Health Care Plans and/or SEND, Looked After in Education children and those that reside in a domestic abuse refuge.

Poverty-proofing is a nationally recognised tool, developed by Children North-East [28], to identify the barriers children living in poverty face in engaging with education, services, activities and opportunities. It was felt that whilst there is increasing awareness of poverty-proofing, programmes like HAF continue to identify and treat differently those who are living in poverty: *“The whole ethos of poverty proofing is about that no activity excludes*,* identifies*,* treats differently or makes assumptions about those because of resources.I think that the holiday activity funded programme could do with being poverty proofed”* (Provider 10). Poverty proofing is a useful tool to understand the ways that interventions like HAF unintentionally promote stigma and can leave families with unexpected costs related to their involvement. There is, however, also a need to consider the wider causes and effects of poverty in relation to the health and wellbeing of families and how this may affect their ability to engage with programmes like HAF.

Some participants wanted to see a more universal and inclusive HAF that enabled children to build social bonds with other children from a variety of backgrounds:

“*I think it should be open to everybody*” (Provider 12)

*“If we can identify additional funding that supports a universal offer so there is no stigma attached to accessing the HAF programme and that actually it’s about holiday activities…irrelevant of you know cost*,* place*,* culture*,* identity*,* etc. that’s really important”* (HAF Lead 4)

Others were hesitant around the idea of a universal offer and noted that there is already a struggle to engage those children eligible for FSMs: “*At the moment we’re reaching…17% of our free school meal cohort”* (HAF Lead 1).

##### Reach

Ensuring eligible families are aware of HAF and can attend, was a particular issue for delivery staff. There is very limited understanding of HAF as a national programme, even for families that already attend: “*It’s still not very well advertised…that this is available and how it’s available”* (Parent 5). Parents and children tended to identify more with the name of the provider or used general terms such as “*Play Scheme*” (Parent 7) to describe the activities their children had taken part in. HAF Leads felt they could be more strategic in their approach to marketing; investing in advertising, branding activities in different ways (e.g. for older children or children with SEND) and having clearer communication around eligibility, combined with more targeted approaches e.g. through HAF champions within schools.

Children also wanted to see better advertising of the HAF programme by, for example, making use of billboards and posters, but more specifically, they wanted to see more information about the types of activities on offer as they thought this would attract more children: *“On an advertiser you could tell them what type of things you do each day. They might think I might want to go then because I like that type of stuff”* (Focus Group 1, Child 3).

Strategies to reach eligible children came with the risk of stigma that made it a challenging balance with the benefits of advertising the programme. Some participants involved in the planning and design of HAF cautioned against the use of language around the eligibility criteria, poverty and hunger and expressed concern that it may affect children’s self-esteem and aspiration in the longer term:

*“That’s why they changed the name*,* [from] holiday hunger”* (Political Lead 3)

*“Part of me that worries that we are continuing that stigmatisation of certain groups of children*,* you know*” (Political Lead 5)

##### Attendance

Attendance was the most common issue mentioned by HAF delivery staff. Low attendance partly resulted from the eligibility and reach challenges described above, but there was still a sense among participants that even if families and children are aware they are eligible, getting children through the door is difficult. Attendance issues that providers faced were: (1) not signing up at all or children attending once or twice and then not returning, and (2) families booking places and then not turning up on the day, leaving them with multiple unused places. For the first issue, the accessibility of the booking system and HAF clubs were important factors. The location was a particular issue when the cost of transport was prohibitively expensive, for families living in rural areas with limited and irregular public transport links and for families with a child with SEND: *“There was a parent that*,* bless her*,* would like get the bus for an hour to drop her kids and then she would just hang around…by the time she got home it’d be time to pick up again”* (Provider 3). Location and transport challenges can be compounded by the lack of flexibility in the start and end times of some clubs, making it difficult for some families to arrive in time for drop off and pick up: *“I know sometimes the timings don’t work. So*,* like*,* it could be like 9 o’clock somewhere*,* and then you’re going to the other end of the city so you’ve gotta get up at crack of dawn”* (Parent 3). For children, not knowing anyone or having negative relationships with other children who attended the HAF club was a reason not to attend (or re-attend): *“Sometimes what makes me not want to go is that sometimes some of the kids can be a bit annoying to you”* (Focus Group 1, Child 3). Whether they were interested in the activities on offer and felt comfortable in the setting were also important considerations. Some children said they may prefer playing out with friends or relaxing at home during the holidays: *“If I saw something on*,* like swimming and if I didn’t go*,* it’s probably because either um I was playing out with my friends or I was with family sometimes”* (Focus Group 2, Child 3).

A variety of reasons were given to explain why some families book their children on to HAF clubs and then do not attend. These included unforeseen circumstances such as sickness or bad weather, not being aware about how to cancel a booking when circumstances change, as well as a lack of value placed on the programme because it is seen to be “free”: “*The problem with offering something for free is that people won’t turn up”* (Provider 12). Providers, HAF Leads and parents talked about the impact these ‘no-shows’ had in terms of financial losses, food waste and taking spaces that could be used by other children: “*What a lot of parents don’t realise…if you don’t go*,* another child misses out”* (Parent 5). To mitigate the impact of no-shows, some providers would overbook places, operate waiting lists and offer rewards or recognition for repeat attendance, but there was widespread agreement that these were not perfect solutions and in fact came with other issues (e.g., children having to be turned away from an overbooked activity).

#### Theme 2: the importance of relationships

The data in this theme primarily came from HAF delivery stakeholders, with some insights from parents. The participants did not mention relationships with the *children to a great extent* (referring instead to ‘families’) and the children did not refer to relationships, although they were not asked directly about this topic.

##### Relationships with families and communities

Trusted relationships with families and communities are key to the success of the HAF programme: “*the strength in the programme is the community and voluntary sector”* (HAF Lead 5). Relationships with families are established by the providers through prior knowledge and engagement with communities, collaborating with schools and other stakeholders, and providing a safe and fun environment for the children that means they want to return. Participants were clear that the HAF model works better in areas where there is already a strong community infrastructure: “*I was someone who worked in the community for like five years. So I knew a lot of people*,* I held a lot of relationships with the children and the young people and the families….And I think that it worked really well”* (Provider 11). Conversely, providers that were less embedded in community, without existing relationships with families, were seen to be less successful: “*that’s why we use community organisations because [they know] families and the communities that they’re working with…and our national providers don’t get that. Our national providers don’t get as much take up as our community providers”* (HAF Lead 4).

HAF Leads and Providers perceived that initial engagement (i.e. during recruitment and enrolment stages) was greater when families were already familiar with the provider organisation and/or delivery staff. For example, parents already had a high level of trust in HAF clubs delivered through schools, and school staff had existing knowledge of the families to target and strategies to engage with them: *“the main thing for me was I was dropping my daughter off to a place that she knew*,* so it’s her school*,* I knew it was in gated grounds*,* I knew I could trust the guys”* (Parent 6). Building relationships was key during the referral process to give first-time attendees the support and confidence to get ‘through the door’, as well as being important for maintaining ongoing engagement and return attendance. This was a pattern that ran through data from HAF delivery personnel, parents and children. When providers know families well, they are more likely to understand the barriers they may face in accessing holiday provision and be able to work with them to overcome these barriers: *“they [club staff] were driving…to pick up kids”* (HAF Lead 2). Parents felt it was important that HAF clubs were perceived as safe, welcoming and supportive spaces for parents and children, with one parent relaying their anxiety about attending new places: “*having that safe space for children to be…making it comfortable and warm and inviting are kind of the most important things”* (Parent 9).

##### A partnership-based model

On a more operational level and representing data from HAF delivery stakeholders only, relationships across LAs, HAF Leads, Providers, and wider stakeholders are vitally important. HAF provision was perceived to be most effective when delivered as part of a partnership-based model i.e., through organisations working together across the sector. HAF Leads and providers formed relationships with a wide range of organisations who could be involved with funding, sponsorship, referring families into HAF, and programme delivery, including delivery of one-off sessions. Survey respondents provided examples of partnership working, such as collaborating with university nutrition students for reviewing food hygiene certificates and EHO ratings.

One provider described successful partnership working, led by a designated partnership manager: “*The idea with having the partnership manager was to kind of coordinate all of the activity that was happening within that geographical area…to help streamline it”* (Provider 10). Another provider relayed the benefits of delivering HAF in a multi-agency centre: “*Just by the fact that we based it in the centre*,* education*,* health and social services were all involved”* (Provider 5). Close partnership working with other sectors helped refer families into the programme and was also useful for signposting families, if further support was needed.

Fostering a strong network of local HAF Providers created a platform for support and enabled providers to share knowledge, particularly when organisations had specialist expertise: *“We’re very much learning from other providers as well…and using those as options to*,* to share good practice…”* (HAF Lead 2). It also developed an awareness of local provision, supporting HAF Leads to streamline available services, and allowing providers to refer families elsewhere if they were unable to meet their needs. Areas which did not have a strong local network suggested this as an area for improvement.

#### Theme 3: delivering a high quality, inclusive HAF programme

All participants involved in the delivery of HAF spoke of the efforts to ensure that the programme was of high quality (e.g., in terms of activities, food and experiences), sustainable (i.e., that LAs and communities could continue to run holiday clubs even if HAF alters or funding stops) and inclusive (e.g., of all children in their community, with particular reference to children with special educational needs and disabilities [SEND]). Data from all participant types, including children and parents, informed our interpretations. There were several patterns across the data, although at times conflicting perceptions, around how best to maximise the funding and what is considered ‘high quality’ and of real value to families and children.

Participants expressed differing views on provision structure and what a ‘high quality’ HAF club should look like. Some HAF Leads and Political Leads highlighted the need to have unstructured provision: “*our forest schools are also really well-attended so anything which has yeah*,* using woods*,* woodlands*,* things like that*,* that very flexible informal approach is really well-attended”* (HAF Lead 11). Contrarily, most providers and some Political and HAF Leads stated that structured provision helped providers, children and families know what to expect and what is going to be delivered: *“I think structured*,* the children know what they’re going to do before they come to you. So*,* a clear sense of this is what’s going to happen. Behaviour expectations and boundaries set out”* (Provider 4). Participants across groups thought HAF was a good opportunity to provide children with new experiences: *“You’re able to get out and like go to places where you usually don’t go”* (Focus Group 3, Child 4). Some HAF delivery stakeholders saw the benefit in trips, but found them to be more expensive and mean they could reach fewer children with the programme: *“The day trip*,* the trip to the seaside*,* the theatre trip*,* the bowling trip on a Friday*,* those are the bits that have been eaten away because there isn’t just enough money in the kitty now from a grants perspective”* (HAF Lead 5). The divergence in the data related to HAF club structure reiterates the need for a community-centred approach that allows delivery stakeholders to be flexible to their capacity and resources as well as the needs and desires of the children locally, as one style of HAF club would not be suitable in all settings and communities. Moreover, many participants - including parents - expressed a need for greater family involvement in decision making to support high-quality, enjoyable, well-attended provision: “*It has to be what the parents need*,* what the kids need*,* and not what someone else decides”* (Parent 5).

Another area of focus, unsurprisingly, related to the provision of high-quality food during HAF clubs: “*I think the food part of it was a real challenge*,* like in being able to find the food*,* provide the food and make it be healthy*,* nutritious and*,* you know*,* within budget as well.”* (Provider 10). Many interviewees shared the same goal of wanting to provide children with healthy food, and if possible a hot meal, in a dignified way. Barriers to offering healthy food at HAF clubs included: insufficient funding (essentially reduced due to cost-of-living crisis); education and certification/experience of providers and them being able to meet School Food Standards within budget; a food culture offering ‘unhealthy’ food as a reward to children; facilities (such as visiting an external venue with no kitchen facilities); and children and families’ feelings, preferences, and expectations around food: “*Some parents did complain it wasn’t chicken nuggets and chips*,* but I tried”* (Provider 4). This final barrier presented a conflict for many participants as they wanted children to eat healthy food, but if children “*won’t eat*” (HAF Lead 8) what they provide, they felt uneasy about sending the children home having not eaten anything: “*We do get providers that put on very healthy food like carrot sticks*,* fruit*,* salads and the kids won’t eat it*,* so what do I do? I don’t want to send them home hungry”* (HAF Lead 3).

Clubs used different techniques to try and tackle the issue of children’s food preferences, with some ensuring variety of food, or flexing the guidelines to prioritise the children eating, even if that was not always considered ‘healthy’: *“they always have backups so they had ham wraps…just in case you didn’t like it*” (Child 3). Others made efforts to involve children in the preparation of food and encouraged them to try new things: *“Sometimes we got to make our own food as well*,* help do it and it was really fun”* (Child 1). However, all creative attempts to ensure a healthy meal were restricted by costs: “*It’s quite hard to do a decent meal for £5 with a dessert and drink”* (Provider 4). It’s worth noting that several participants (both HAF delivery staff and parents) felt that the food element of HAF was not the most crucial part of the programme and it was not parents and children’s top priority when attending: “*I think the food is a bonus for lots of children*,* but I don’t think the food is the focus for…any single child that attends the HAF”* (Parent 5). This highlighted debates about what HAF is trying to achieve and whether it has too many objectives with limited capacity.

Across all participant groups, the challenge of making HAF inclusive of children with SEND was highlighted. Difficulties in inclusive provision related to the additional resource, capacity and training (and therefore HAF funding) needed to meet the needs of children with SEND: “*We’ve got a SEN specific offer now*,* but it’s costing them a lot of money*” (Parent 5). Further, there is complexity surrounding the range of different needs children may have, with there being desire to have some specialist provision but also to ensure that ‘mainstream’ HAF clubs are suitable for children with SEND should they wish to attend. The range of need and lack of training often meant that clubs lacked clarity in their advertising regarding the accessibility of the activities, food and environment, which families of children with SEND found difficult to navigate and sometimes led to exclusion from HAF. The data were so rich and policy-relevant related to provision for children with SEND that we chose to explore this in a separate forthcoming paper, so we only highlight it briefly here.

Finally, in terms of sustainability, HAF Leads were keen to build capacity and upskill local organisations and communities: “*there’s a legacy*,* ultimately*,* for organisations to continue to work with families*,* long-term.”* (HAF Lead 7), which was partly about futureproofing should *“the Department for Education funding not be around.*” (HAF Lead 6). This was also thought to improve the quality of HAF provision being delivered. Each region adopted different approaches to building capacity. For example, one LA worked in collaboration with a third-sector partner to support voluntary organisations delivering HAF. Another area had a young leaders programme, equipping young people eligible for HAF with the knowledge and skillset to deliver future HAF programmes: “*they work at HAF camps and some of them when they reach an age they’re remunerated for that and they become part of the staff. So they go through training and so on”* (Political Lead 1).

#### Theme 4: schools as trusted advocates

Effective partnerships with schools were seen as key to the success of HAF. There have been a number of models that have been developed in different LA areas, for example some HAF Leads predominantly work with schools to identify children who would benefit from the programme and to support signposting, with some building networks of *“HAF Champions”* (HAF Lead 1) within schools. Others have focused on building partnerships between schools and community providers and making use of school staff and facilities. Political and HAF Leads felt that schools make excellent providers, as they have the facilities (e.g., halls, sports fields, kitchens), skilled staff and procedures (e.g., safeguarding, health and safety) required to deliver high quality, low cost, accessible provision. The familiarity and safety of schools was highlighted by some providers and parents as an important factor in contributing to attendance. However, we interpreted a level of complexity with school involvement in terms of hosting clubs as several parents wanted their children to be in new environments during the holidays (outside of the classroom). Further, other parents highlighted that many children do not want to be in a school setting during the holidays, with some finding it uncomfortable, particularly if they don’t enjoy being at school: *“My child’s had quite a complex history*,* a lot of school trauma. The [HAF] project is actually based in early years provision so it’s an old school building. He was quite triggered by it”* (Parent 5).

Regardless of whether schools were involved in delivering HAF provision, there was widespread agreement that they play a key role in identifying and signposting eligible children to HAF. Parents and children noted that schools are a trusted source of information, and they agreed that the best way for more families to find out about HAF was through schools. Schools are best placed to identify children who would benefit most from HAF, including those who are at risk of exclusion and persistently absent from school. In some areas, this signposting is very effective with school staff, who know children and families well, supporting them to sign up and take part in HAF. However, most Political and HAF Leads spoke of challenges around school engagement in the programme: *“We don’t have the extent of the engagement in schools that would be good. But then you see…schools are battling with their own problems…funding*,* behaviour*,* COVID*,* pay…but yeah*,* it would be good to have more schools involved”* (Political Lead 4).

Participants felt that there is a need to understand and articulate how the involvement of schools in the HAF programme might be mutually beneficial (e.g. to support the attendance, educational attainment and safeguarding of disadvantaged children) and how to maximise the benefits of their involvement without negatively impacting on already stretched staff and resources. Participants felt that more consistent and formalised school involvement in HAF would also mean that, rather than a standalone programme, HAF is embedded in children’s lives. *“Are we seeing the impact of when kids go back to school? That doesn’t feel joined up terribly well to me” (Political Lead 2).* While this may enable better communication and multi-agency working from the perspective of HAF delivery staff, as our parent quote above shows, there is a level of complexity that should be acknowledged in relation to schools’ involvement. Specifically, that the separation of HAF clubs and schools might be preferable for some children and families who have a more difficult relationship with schools.

#### Theme 5: evaluation of HAF

Providers and HAF Leads felt that the mandatory HAF evaluation did not represent the full impact of HAF. This was partly because Department for Education data collection is focused on the reach of HAF, and reporting raw numbers does not capture the nuances of delivery (e.g., differentiating between high attenders and those who had only participated in one session of the programme). As a result, while findings are useful in evidencing what had been delivered, they have little value for informing future delivery.

Alongside mandatory data collection, many areas conduct their own evaluations of HAF delivery, both on a local area and individual provider level. Some areas also commission external evaluations, for example, partnering with universities. Additional data collected included satisfaction ratings, financial returns, and qualitative feedback from providers, parents and children. Case studies were also perceived to be particularly important for capturing individual impacts and the longer-term ‘ripple effects’ of HAF, including the likely social return on investment. The main purpose of these additional evaluations was to assess the impact of HAF and to reflect on current provision. Findings were used for marketing and promotion activities, to demonstrate the impacts of HAF to funders and the Department for Education, and to improve future delivery: *“We rely heavily on demonstrating our impact to people that fund into us so that we can sustain the activity.”* (HAF Provider 1). Informal daily feedback was also important for informing delivery and supporting staff morale. This was achieved by direct feedback from attendees and through conversations with parents at drop-off and pick-up. One parent relayed that they had never been asked for feedback on HAF delivery, suggesting that the type and extent of evaluation varies between clubs and areas.

## Discussion

A major finding of our research was that one of the greatest challenges for HAF delivery stakeholders is in navigating the eligibility criteria, reaching eligible children and ensuring high attendance at HAF clubs. The findings of this study raise questions regarding the appropriateness of the FSM threshold – which has not been updated since 2017. As well as causing operational challenges for those delivering HAF, approximately 1 million children living in poverty are excluded from the support of school meals and HAF provision [29]. The criteria also exacerbate stigma for eligible families [3, 30], which acts as a barrier to access [29, 31].

There was a pattern within our data across participants and contexts that highlighted the value of flexibility, agency, and community in tackling food insecurity and improving health. Similar findings have been reported elsewhere; for example, an evaluation of the Food and Fun clubs in Wales reported that flexibility is a key ingredient to ensure holiday provision is popular with families, can be implemented at low-cost and provides health and education benefits [32]. Moreover, stakeholders have advocated for a flexible model of holiday provision that uses and develops existing community assets [3], and the rigidity of the Department for Education HAF guidance has been identified by LA staff as a barrier to programme implementation [33]. Furthermore, staff from holiday clubs in England and Wales have expressed desire for more autonomy over the provision they deliver [22].

While previous studies suggest that the importance of these components is understood at a public, practice and local policy level, we argue that there needs to be greater recognition from national policymakers of the necessity of embedding flexibility, agency, and community within provision. For example, through facilitating supportive policy and funding for community organisations to have agency, choice and flexibility to decide how best to work with their families, without the constraints of rigid HAF criteria. The findings of this study denote that this would result in high family engagement, as community organisations are trusted, non-stigmatised, and could offer open-access provision across the whole year that responds to local wants, needs, and resources. Flexibility must be balanced with quality and evaluative frameworks must be in place, particularly as the findings from this and other studies [34] suggest that some HAF Providers are not delivering food that meets the School Food Standards. Therefore, while there is a strong case within our data for greater flexibility, there is then the potential risk of even wider variation in the quality of provision, which is not necessarily responsive to families and communities, and it difficult to evaluate.

A novel finding of this study is that HAF staff and eligible families feel that the HAF programme is not poverty proofed [35] or adopting a poverty-informed approach, i.e., it does not always account for the significant barriers to attendance for families experiencing poverty, despite the clubs being free. Accessibility issues in this regard were raised relating to all aspects of the programme, including the eligibility criteria, methods used to reach and sign-up families, and the provision itself (e.g., location, food and physical activity provided). Moreover, inequalities are embedded within these accessibility issues. Children and families who: live more rurally, for whom English is not their first language, or who have additional needs, were seen to be disproportionately affected. Previous research highlights further inequalities; while 47% of children from Black and minority ethnic groups are in poverty, compared to 24% of white children [1], holiday clubs are less likely to be situated in economically disadvantaged areas with a higher proportion of people who are Black and from minority ethnic groups [36]. Further, there is evidence that implementation stakeholders of holiday programmes report that families with disabilities, as well as Black and minority ethnic groups, are underrepresented within programmes of holiday provision, even within universal provision [3].

The findings related to the accessibility of HAF are concerning, not least because young people experiencing intersectional disadvantage have identified local community spaces as one of the few places they feel safe to be active [37]. Flexibility and choice were again perceived to be key to resolving many of these access issues to HAF; for example, adopting flexible eligibility criteria, offering various sign-up methods (e.g., online, offline), allowing flexible drop off/pick up times or drop-in sessions, and providing multiple physical activity and food options to suit varying child needs and preferences, which the community organisations are already familiar with. Ultimately, HAF needs to be more flexible and responsive to the needs of the families it is designed to support. Having a knowledgeable, able and confident workforce who the families trust was seen as essential to this, with adequate funding and training being necessary predecessors.

We identified that there is considerable complexity and divergence of opinion around the role of schools in HAF. As in previous research [22, 32], schools were highlighted by some participants as valuable settings for holiday clubs, as they have the necessary facilities and are likely to be more familiar and accessible to local families. Some participants, however, particularly HAF Providers and parents, emphasised that schools may not be an appropriate or supportive setting for many children and families, as not all children will view school as a safe environment. Additionally, participants cited poor engagement from schools in HAF, due to logistical and capacity issues, which corroborates with previous research [38]. Where opinions of participants converged was around the value of schools as knowledgeable and trusted sources of information who can identify families and signpost to local clubs best suited to them. We must caveat this by noting that some children who are eligible for HAF may not be attending mainstream school [39] and for some regions and activities, schools may be the most appropriate club host; it’s thus essential that schools are just one part of a multi-dimensional strategy to support families during the holidays. Future research is also needed to explore the perceptions and experiences children not attending school, as they are underserved and underrepresented in research [40, 41].

The economic survey results highlight the variation in HAF implementation and delivery across the surveyed LAs. However, consistent inclusion of core elements such as food, physical activity, enrichment activities, and nutrition education reflects robust adherence to the programme’s objectives. Notably, many LAs have gone beyond the Department for Education’s requirements by, for example, continuing to offer food education for families and carers, despite this element being dropped from the guidelines. We also identified variation in the grant amounts received from the Department for Education and in the availability of matched funding. It is important to note that funding is largely dependent on the LA size and the number of HAF-eligible children, which were not accounted for in our analysis. Furthermore, little information was provided on the amount of matched funding. Therefore, we were not able to calculate the funding amount and cost per child. Our results highlight a number of challenges including the reliance on external funding to sustain and enhance the programme and over-subscription, which indicate the need for developing a more sustainable funding model to support the delivery of HAF programme across England. The provision of subsidised attendance for vulnerable and ineligible children illustrates the willingness of LAs to enhance the programme’s inclusivity, yet it also points to the systemic gaps that necessitate such measures (Table 2).

**Table 2:** Policy, practice and research implications of our findings

Finding	Policy & practice implications	Research implications
Challenges navigating eligibility criteria, reaching children and club attendance	*For HAF*: • Reconsider FSM as eligibility criterion. • Allow additional funds to enable marginalised families to attend. *Wider food insecurity policy*: • Strong engagement/co-production with families who are both eligible and not eligible for FSM. • Consider FSM provision for all children in schools, taking direction from the current intervention on this within London.	• More in-depth analysis of the reasons why eligible families are not attending HAF, and what else they would benefit from in the school holidays otherwise.
Flexibility, agency and community crucial to the running of HAF	*For HAF*: • Funding community organisations to deliver HAF, being led by the diverse needs of the micro and hyperlocal community (flexible and not too prescriptive), building strong relationships within communities, and linking community organisations to support their work in and outside of HAF. • Need and desire to be flexible with how guidelines are interpreted and implemented to suit the local context and local priorities/values, support a diverse range of providers, and improve reach and attendance. In particular, flexibility around timing and duration of provision and the food element. • Family involvement in decision making. • Local HAF teams offering choice in activities and food options. • Importance of flexing guidance in response to feedback on what is working and not working on the ground. Ensuring ‘programmatic memory’. *Wider food insecurity policy*: • Piloting ‘Cash First’ approaches as alternatives to food parcels to increase dignity and agency, and a longer-term solution to support families out of poverty.	• Testing of evaluation methods for flexible and community-centered food insecurity interventions. Evaluation of flexible, community-centered approaches (e.g., providing funds to community groups with little restriction) for child health outcomes.
Families find it a challenge to access HAF	*For HAF*: • Clearer, more transparent, targeted marketing of the programme – what HAF is, who it is for, it’s value, range of provision on offer, accessibility of clubs. Non-stigmising language and minimise concern of hidden costs e.g., ‘fully-funded programme and Department for Education covers cost of activities, transport, food’ ‘You don’t have to bring anything and you don’t have to pay anything’ • All branding and marketing resources in multiple languages, using videos to share key messages, and alternative platforms e.g., WhatsApp • More options regarding signing up including paper versions and a sign-up/booking support service. • Local HAF teams to provide transport to clubs and/or subsidised travel to a club on a public transport route. • Offering flexible drop-off and pick-up times. • Training for all HAF staff on inclusivity and accessibility practices, and specialist training in supporting children and families with SEND. *Wider food insecurity policy*: • Poverty proofing • Consultation and co-production with families • Funding allocation for reach and recruitment	• Investigate barriers to accessing HAF with children and families not currently attending. Are these insurmountable and should another type of intervention (e.g., cash first approach) be considered. • Extend research to understand the barriers to accessing food insecurity interventions for those experiencing intersectional disadvantage, particularly for children with SEND or those from ethnic minority backgrounds.
School involvement	*For HAF*: • Formalise school involvement in relation to signposting and reaching children who would benefit from HAF. • Provide opportunities for school staff involvement in HAF delivery to capitalise on existing relationships with children and families. • Consider schools as a possible venue for HAF clubs, but ensure community groups are also strongly represented. *Wider food insecurity policy*: • Enhance collaboration between schools, community groups, LAs and Government to reduce food insecurity.	• School involvement in child health, poverty and food insecurity policy and interventions. • How best to support children experiencing poverty not attending mainstream education.
Need for comprehensive evaluation	*For HAF*: • Continual reciprocal local evaluations. Adapt provision in response. • Involve families in evaluation. • National ripple effects mapping evaluation capturing voices of children, families & communities.	• Better understanding of what works within different delivery contexts (e.g. secondary age provision, SEND) • Studies exploring longer-term outcomes/impact of HAF, both for children and families, and for communities.
Funding and sustainability	*For HAF*: • Joined-up working within the LA and the wider community. • Collaborating with other organisations addressing food poverty to look at addressing the issue at a wider strategic level. • Embed within local strategy across the whole year and as a longer-term investment. *Wider food insecurity policy*: • Comprehensive longer-term strategies to address food insecurity, at national and local authority levels.	• Larger scale return on investment study of HAF to explore case for longer-term investment.

### Policy and research implications 

Our policy, practice and research implications are contained in Table 2.

#### Strengths and Limitations

This is among the first mixed-methods studies to explore HAF provision across different English sites. A key strength is that data were gathered from a range of stakeholders, parents and children across England to gain a fuller picture of what it is like to deliver the HAF programme and what it is like to attend.

One limitation is that the sample does not represent children and families who are eligible, but do not attend HAF clubs. Given that one of the major challenges related to HAF is around attendance, understanding the reasons so many families do not attend is vital for development of the HAF programme. Moreover, we acknowledge that as HAF is a government-funded programme, participants may have been cautious of saying anything too critical. Both parents and stakeholders delivering HAF often caveated their views by stating that they were grateful for the HAF club/funding and would not want it to be taken away. This dynamic will have undoubtedly shaped our data.

While measures were taken to try to achieve diversity in our sample, an open-ended answer option was used on consent forms to record ethnicity and many participants provided nationality, as opposed to ethnicity. As a result we have not reported ethnicity data for our sample. We did achieve some diversity in our sample, but the majority of participants were White British. Future studies (particularly of users of HAF, parents and children) should enhance efforts to recruit those at intersections of disadvantage. Moreover, the small size of the subgroups (e.g., 10 parents, 5 local government leads), meant that some participants had to be in a certain job role or to attend HAF. We were able to recruit representation from children with SEND, but further involvement of children with SEND as well as exploration of more suitable methods (e.g., creative, non-traditional methods) is needed in future research so that more children can meaningfully contribute.

## Conclusion

Our findings reveal a range of opportunities and challenges for the delivery of school holiday provision of enrichment activities and food targeted at low-income children, such as HAF. To ensure that low-income families are receiving the support they need during school holidays, we recommend that the government implement policy and funding for community organisations to have agency to decide how best to work with their families, achieved by greater flexibility within the national guidelines and criteria. Moreover, we recommend *poverty-informed*,* place-based* holiday provision that is iteratively developed with the involvement of local families. Incorporating comprehensive evaluations (beyond monitoring attendance) will contribute to debates around HAF provision and the extent to which it is positively impacting on children’s health and wellbeing, physical activity levels and school readiness.

## Supplementary Information

Supplementary Material 1.

Supplementary Material 2.

Supplementary Material 3.

Supplementary Material 4.

Supplementary Material 5.

Supplementary Material 6.

Supplementary Material 7.

Supplementary Material 8.

Supplementary Material 9.

Supplementary Material 10.

## Data Availability

The datasets supporting the conclusions of this article are available in the University of Bristol data archive: https://data.bris.ac.uk/data/.
