# Supplementary material for: Perceptions of the national school holiday programme (Holiday Activities and Food) in England as experienced by local government, service providers and users of the service: a mixed-methods study
Source: BMC Public Health. 2026 May 30;26:2425. doi: 10.1186/s12889-026-27699-1 (PMC13501641; doi:10.1186/s12889-026-27699-1)
Supplement: Supplementary file 1 [file 12889_2026_27699_MOESM10_ESM.docx]

**Additional File 10: HAF health economics survey summary**

**Methods**

The economics survey was developed by the study team based on the objectives of the study. The aim of the survey was to understand the nature of HAF programme delivery across the country, including how the programme is financed, and what costs are associated with its delivery across the participating local authorities. The survey specifically focused on HAF provision during the summer 2023. The study health economist (IP) developed the initial version, which was reviewed by the study team. The survey was sent out to eleven HAF Leads via email.

**Results**

Nine HAF Leads completed the survey.

***HAF provision***

19 different types of providers were mentioned by the respondents to be involved in the HAF programme delivery in the surveyed local authorities (LAs). Schools, charities, youth clubs, community clubs, and churches/faith groups were the most common providers.

| **Type of provider** | **N** |
| --- | --- |
| School | 9 |
| Charity | 9 |
| Youth club | 9 |
| Community group | 9 |
| Church/faith group | 8 |
| Sports club/provider | 6 |
| Nursery | 5 |
| College/university | 4 |
| Leisure centres | 4 |
| Housing association | 2 |
| Community interest companies | 2 |
| Independent arts & culture providers | 2 |
| Out of school franchises | 1 |
| Children’s centre | 1 |
| Local authority services | 1 |
| Learning providers | 1 |
| Family hubs | 1 |
| Small business food providers | 1 |
| Large business food provider | 1 |

Nine respondents reported that in their LA, the HAF programme was delivered according to the core offer requirement, which is four weeks of face-to-face provision covering a minimum of sixteen days. For LAs that have a summer holiday less than six weeks, the minimum requirement is twelve days over four weeks. Four respondents reported that in their LA the provision exceeded the core offer requirement with provision spread over a greater number of weeks (n=2) or provision for over 16 days (n=1).

| Met core offer requirement | Yes | 9 |
| --- | --- | --- |
| Exceeded the core offer requirement | Yes | 4 |
|  | No | 5 |
| How the core offer was exceeded | Provision spread over 6 weeks instead of 4 | 2 |
|  | Provision for over 16 days | 1 |
|  | Unclear | 1 |

**Food, enrichment and physical activity**

Food, physical activity, enrichment activities, and nutrition education were provided in all LAs that were surveyed. Seven out of nine LAs provided an enhanced food offer beyond one meal a day by, for example, also offering healthy snacks or a second meal. In four LAs, there was also an enhanced physical activity offer of, for example, sessions running longer than 60 minutes. The types of physical activity offered included sports; play; sessions at adventure playground sites that include climbing, play, running, bike trips, football tournaments and hikes; dance; golf; and tennis.

| **Type of activity** | **N** | **Specification achieved** | **N** |
| --- | --- | --- | --- |
| Food | 9 | Minimum required specification | 2 |
|  |  | Enhanced offer (e.g. healthy snacks, more than one meal per day) | 7 |
| Enrichment activities | 9 | N/a |  |
| Physical activity | 9 | Minimum required specification | 5 |
|  |  | Enhanced offer (e.g. sessions longer than 60 minutes per day, wide range of activities on offer) | 4 |
| Nutrition education | 9 | N/a |  |
| Food education for families and carers | 7 | N/a |  |
| Signposting | 9 | N/a |  |
| Other | 2 | N/a |  |

Providers offered a variety of enrichment activities, including:

- Cooking, baking, gardening, sailing, rock climbing, swimming, crafts, dance, drama, forest school, trips to historical attractions and nature reserves, residentials and sports clubs, arts and culture, gym memberships, specialist provision for certain groups, parks programme, forest school, dino digs, puppetry, performing arts, equine husbandry, African drumming, circus skills, music, t-shirt printing, photography, theatre, STEM activities, programmes to enhance learning, digital, and entrepreneurial skills.

In one LA, enrichment activities were provided to whole families allowing them to attend cultural, education, and social events.

*‘Without the HAF programme many would not get the opportunity.’*

Nutrition education, in the form of cooking sessions, conversations about nutrition, and links to local food pantries and food providers, was also reported to be an important component of the HAF programme delivery.

*‘Each delivery period we offer the children’s kitchen sessions to providers. These are workshops where children make their own lunch. This process allows providers to build confidence and start to make lunches with the children themselves.*’

Nutritional information and education were provided during meal times in some LAs.

Provider support and education was mentioned to be an important element of nutrition education.

‘*We also offer food leaders training to all providers. This is a 5 day accredited course to train providers to cook in batches, hold cooking sessions and bring food into their provision in a legal and safe way*.’

Furthermore, in one LA, portable cooking kits and training on how to use them were provided for the venues that had no cooking facilities available.

While food education for families and carers is no longer included in the DfE guidance, seven out of nine LAs reported providing this activity. This included cooking sessions with parents, providing take home recipes and food parcels linked to what the children made that day, providing public health information on healthy eating.

Many providers, particularly voluntary and community organizations, also offered signposting via newsletters, direct communication with parents, school roadshows, and provision of information and literature. One respondent reported that signposting was more challenging for private providers:

*‘When we work with private organisations, they sometimes find it more difficult to know what to signpost to and for this we use our website and other steering group members for support.’*

***HAF funding models***

All respondents provided information on the amount of funding received from DfE to deliver the HAF programme in the summer 2023. The mean range of the grant was £1,546,800 ranging between £200,300 - £5,000,000. In 3 LAs, LA/council also provided financial and/or in-kind support. Housing associations, universities, food providers, local businesses, supermarkets, and third sector organizations were also mentioned by some respondents as providers of financial and in-kind support. In fact, additional sources of funding and support were reported to be a crucial element in the delivery of HAF:

*‘All HAF providers are encouraged to seek match or in-kind funding to extend provision. Providers share at point of application the total project costs and the amount requested as a HAF grant. This additional match/in-kind funding is used to create further spaces for children and to add further value to the existing programme.’*

*‘Some delivery partners secured matched funding amounting to £141,100 from a*
*variety of sources including housing providers, other grants and internal staffing*
*costs. This was used on a range of things including additional staffing and resources.*
*A number of groups also used volunteers to support with the delivery, administration*
*and management of their HAF project.’*

Some funding issues were also indicated. The respondent from one LA reported:

*‘The HAF programme in [City] is very large and diverse. We were, however, significantly over-subscribed for summer grant funding and as such not all providers were funded, or funded to the level they necessarily anticipated. Priority was given to districts based on FSM eligibility level and sufficiency, meaning that those areas previously benefitting from previous ‘underspend’ of other areas were not funded as in previous periods. This did create some challenge and push-back from providers, ideally we would like to be able to fund all viable projects that apply for grant funding.’*

| **Funding source** | **N** | **Mean (range)/type of support** |
| --- | --- | --- |
| DfE | 9 | 1,546,866 (200,373 – 4,724,352) |
| Out-of-pocket fees from ineligible participants | 0 |  |
| LA/council | 3 | 450,000  In-kind support, household support funding |
| Housing association | 1 | 1,500 |
| Universities | 1 | In-kind support |
| Philanthropists | 0 |  |
| Sponsors | 0 |  |
| Food providers | 1 | 2,500 |
| Supermarkets | 1 | ‘Unknown as many of our local groups get reduced food costs donations. Not recorded by HAF’ |
| Local businesses | 2 | ‘Asda, Tesco, Morrisons, Sainsbury’s and many local suppliers provide cash, vouchers and provisions. As above we do not ask our providers to record this.’  Building Society - 100000 |
| Other | 1 | ‘Some delivery partners secured matched funding amounting to £141,072 from a variety of sources including housing providers, other grants and internal staffing costs. This was used on a range of things including additional staffing and resources. A number of groups also used volunteers to support with the delivery, administration and management of their HAF project.’ |
| Third sector | 4 | £325,000 |
| In-kind donations | 4 | E.g., Donations from bakery |

***Cost of HAF programme delivery***

The mean cost of HAF programme delivery was £1,480,296 ranging between £400,000 and £4,724,352, depending on the LA size and the number of children attending. Six respondents also reported the staff costs associated with the HAF programme delivery, which were £64,543 on average (range £26,469 - £106,000). One respondent provided the breakdown of the following breakdown of the staff costs:

- ~£10,000 HAF Coordinator
- ~£3,500 Project Support Officer
- ~£13,000 Steering group support (quality assurance visits and project strategic support)

Furthermore, one respondent indicated that ‘a maximum of 10% of the budget can be used for council management costs. The cost of staff for our providers is normally around 60% of the budget.’

Five out of the nine respondents indicated that in their LA fully funded or subsidised attendance for ineligible children, including:

- Children with Education Health Care Plans, special education needs, Looked After in Education and No Recourse to Public Funding
- Other vulnerable children e.g. those that reside in a domestic abuse refuge
- Young people living in areas of high deprivation who are not eligible for benefits related free school meals and who are not eligible through having other vulnerabilities

In one LAHAF attendance is subsidised for Jewish children:

*‘Following discussions with the DfE we provide funding our Jewish children in [Town]. Their educational sites do not get considered when funding is calculated. We have done some work showing that over 80% of children within the Jewish Community would qualify for Benefits related FSM. We also fill places that our vacant with children identified by partner organisations.’*
