# Supplementary material for: Perceptions of the national school holiday programme (Holiday Activities and Food) in England as experienced by local government, service providers and users of the service: a mixed-methods study
Source: BMC Public Health. 2026 May 30;26:2425. doi: 10.1186/s12889-026-27699-1 (PMC13501641; doi:10.1186/s12889-026-27699-1)
Supplement: Supplementary file 2 [file 12889_2026_27699_MOESM1_ESM.docx]

**Additional File 1: Stakeholders involved in HAF delivery role description**

| **Stakeholders involved in HAF delivery** | **Description of role** |
| --- | --- |
| HAF Political Leads | Local government political role. These participants are elected by constituents to the position to lead on certain activities and services delivered by the local authority (such as HAF) and represent their constituents in the local authority area. |
| HAF Leads | An individual responsible for delivering and managing the HAF programme across their area, including communicating with all the HAF Providers in the area. This includes non-party political civil servants within the local authority and those external to the local authority who have been commissioned by the local authority to coordinate the programme. |
| HAF Providers | An individual who delivers a HAF club either from a community organisation, private entity or school. They will have applied to the local authority for the funds to deliver their proposed HAF club. |
