# Supplementary material for: Perceptions of the national school holiday programme (Holiday Activities and Food) in England as experienced by local government, service providers and users of the service: a mixed-methods study
Source: BMC Public Health. 2026 May 30;26:2425. doi: 10.1186/s12889-026-27699-1 (PMC13501641; doi:10.1186/s12889-026-27699-1)
Supplement: Supplementary file 3 [file 12889_2026_27699_MOESM2_ESM.docx]

**Additional File 2: Table representing HAF Provider club by location context, size, setting and activity type**

|  |  | *n* = |
| --- | --- | --- |
| **Region** | North East | 4 |
|  | Midlands | 3 |
|  | South West | 5 |
| **Location** | Urban | 8 |
|  | Urban-coastal | 1 |
|  | Rural | 2 |
|  | Rural-coastal | 1 |
| **Size** | Large | 5 |
|  | Medium | 4 |
|  | Small | 3 |
| **Setting** | School | 1 |
|  | Community | 11 |
| **Activity type** | SEND-provision | 4 |
|  | Art | 1 |
|  | Sport | 1 |
|  | Mixed | 2 |
|  | Non-SEND provision | 8 |
|  | Art | 1 |
|  | Sport | 3 |
|  | Mixed | 4 |
