# Supplementary material for: Perceptions of the national school holiday programme (Holiday Activities and Food) in England as experienced by local government, service providers and users of the service: a mixed-methods study
Source: BMC Public Health. 2026 May 30;26:2425. doi: 10.1186/s12889-026-27699-1 (PMC13501641; doi:10.1186/s12889-026-27699-1)
Supplement: Supplementary file 4 [file 12889_2026_27699_MOESM3_ESM.docx]

**Additional file 3: Holiday Activities and Food Programme Interview Topic Guide – LA Political Leads**

**Consent**

If a signed consent form has not been returned by the participant, oral consent will be obtained and recorded. In this case, read the consent form to the participant and record as a separate file to the interview.

Participants will then be told about the confidentiality procedures using the following script.

*Before we get started, I’d like to tell you that our conversation today will last no more than an hour and I will be recording the conversation. The recording is to help us remember what you said. You can ask for the recording to be stopped at any point. After we have written a report about all the opinions we have heard here and with other participants, the recordings and notes will be destroyed, so none of the information that is written down and recorded can be connected to you in any way. This means that any names or phrases that you use which could identify you will be coded or anonymised in the transcripts and reports so that you can’t be identified*. *You may decline to answer any question or stop the interview at any time, and you don’t have to give a reason why.*

*Does what I have just read to you make sense? If yes proceed to*

*Are you happy for the conversation to be recorded?*

**Yes?** Proceed to interview.

**No?** Ask if they would like the information read to them again or whether there is anything they would like to be explained further. If ‘yes’ read again or offer further clarification. Then ask again:

*Are you happy for the conversation to be recorded?*

**No**: Thank participant for time and end the interview by informing them that as we are unable to record the conversation, we cannot conduct the interview.

*Thank you for taking part in the interview today. We are interested in your opinions on the most recent Holiday Activities and Food (HAF) programme (i.e. the summer programme) and would like to discuss these with you.*

*This interview will broadly consider the following themes:*

1) *Delivery of the programme*
2) *Reach of and engagement with the programme*
3) *Impact and evaluation of the programme*
4) *Limitations and challenges with the programme*
5) *Best practice guidance*

**Participant Context**

1. Could you briefly describe your role and how it connected with the most recent Holiday Activities and Food programme?
   a. Did you have any direct role in HAF programme policies, commissioning or implementation? Please expand.

**Delivery of the programme**

**Policy, positioning, and planning**

2. Who was responsible for the interpretation of HAF programme policy?
3. Policy context: What was the strategic and political positioning of the programme within the LA?
   a. how did this impact how the HAF standards/guidelines were interpreted?
   b. how did this impact resourcing and provision?
4. Who was responsible for the planning and coordination of the programme?
a. How was demand, supply, and provision identified / mapped?
5. Financial context: How did the model (i.e. sources and mechanisms) of funding:
   a. impact how the HAF standards/guidelines were interpreted?
   b. impact resourcing and provision?
6. How did the funding allocated:
   a. impact how the HAF standards/guidelines were interpreted?
   b. impact resourcing and provision?
7. Moving forward, if funding is reduced or cut how would this influence provision?
8. What existing resources and assets did you have to support delivery of the programme and how did this impact resourcing and provision?

**Provision**

9. What were the key aims, goals and beliefs of the programme?

Cultural context:

a. What are your thoughts regarding these? Are they all equally important?
b. Did you observe any similarities and/or differences between the aims, goals, or beliefs of the HAF programme and those of the HAF commissioners, coordinators, providers, or attendees?
c. How did this impact how the HAF standards/guidelines were interpreted?
d. How did this impact resourcing and provision?
10. How was the HAF programme coordinated and delivered within your LA?
    a. Who delivered the programme, which partners were involved (e.g., LA, schools, external agencies [public/private/voluntary sectors])?
    b. What locations and providers / clubs were used and why?
    c. *(ask if the participant did not complete the survey)* What was the scope of provision (e.g., universal/targeted; core six week offer vs. extended offer; number of days/hours per week; government defined vs. extended eligibility)?
    d. *(ask if the participant did not complete the survey)* What were the components / types of provision delivered?
11. What guidance, resources, and/or connections were used to plan HAF delivery and the elements involved?

Prompts:

a. physical activity elements (e.g., linking with local active partnerships and national governing bodies of sport, using Sport England Strategy on getting YP active)
b. the food-based / healthy eating components
c. other elements/enriching activities
d. parent activities?
e. the food/drink that is served
f. health and safety
g. safeguarding
12. Did provision differ for different groups of children experiencing food insecurity and/or with additional needs)?
    a. How was provision made accessible and appropriate?

**Reach and engagement**

13. How well do you think the HAF programme reached and engaged the families it is aimed at (HAF-eligible families) in your LA?
a. Did it engage children and families that were representative of the ethic mix of eligible families?
b. How did any changes to the eligibility parameters impact reach and uptake/ engagement?
14. How did the social and economic environment in your area influence the reach of and engagement with the programme?
a. level of deprivation
b. number of eligible families
15. How did service and organisational factors influence the reach of and engagement with the programme?

Prompts:

a. size of / structure within the LA
b. type of provider organisation
c. Organisational culture
16. How did financial factors influence the reach of and engagement with the programme?
    a. sources and mechanisms funding for the programme
17. How do you think recruitment of HAF-eligible families could be improved?

Prompts:

a. Using incentives
b. Oversubscribing
c. Working with agencies e.g., child centres, family support workers, social services
d. Social media marketing
e. Avoiding stigma – how?

18. How do you think attendance at sessions (i.e. the number of sessions families attend, returning each holidays) could be improved?

**Impact and evaluation**

19. How was the HAF programme evaluated and how was the information used?
    a. Who was responsible for these processes?
20. Was educational progress as a result of HAF attendance measured?
    a. How was it measured?
    b. What were the successes and ’failures’ or problems and solutions with measuring educational progress?
21. Have you observed any effects (positive or negative) of the programme?

Prompts:

a. Food insecurity
b. Healthy eating
c. Physical activity
d. Fitness
e. School readiness – behaviour, confidence, social skills
f. Parent’s knowledge and skills around buying and making healthy meals
g. Character development (e.g., resilience, confidence, curiosity, honesty, emotional skills, problem solving)?
h. Educational development/ progress
22. How can education progress from participation in the HAF be maximised?
    a. What are the most appropriate and reliable methods of measuring educational progress?
23. What potential do you think the programme has? How worthwhile do you think it is?
24. Were contextual factors (e.g., social/economic, geographical/environmental, cultural, organisational, financial) taken account of when evaluating the programme? If so, how?
25. What opportunities were there for giving feedback on the HAF programme? (at a club level, local authority level, national level) What opportunities are there for sharing experiences/good practice examples etc.?

**Limitations/challenges**

26. Did you experience any challenges, set-backs, or ‘failures’ in developing policy, commissioning and/or delivery of the programme?

Prompts:

a. Marketing/recruitment
b. Staffing during summer holidays
c. School closures for maintenance during holidays
d. Accessibility/transport to available sites
e. Proving daily food for high number of children
f. Quality provision
27. Are you anticipating any challenges moving forward?
28. Were there any contextual factors (e.g., social/economic, geographical/ environmental, cultural, organisational, financial) that you think played a role in the limitations/challenges of the programme?

**Changes to the programme / best practice guidance**

29. What does the programme look like when it’s at its best?

Prompts:

a. Structured / flexible?
b. Location/setting – use of school and staff?
c. Providers/partnerships/networks - multi-agency model?
d. People – community member volunteers, involving young people in design and delivery?
e. Inclusive – involving the whole family, representation from all ethnic groups?
f. Connected – with other initiatives and strategic agencies
g. Sharing of best practice? How?

30. If you were to make changes to the programme, what would they be and why?
a. Do you think these changes would influence the impact of the programme?
b. How feasible do you think it is to make these changes?
31. How can the best practice guidance developed from this project support future policy and improvement of the programme?

**Closing**

*That’s all the questions I have for you today. This has been a really informative interview, thank you. Is there anything else that you think is important that we should know about regarding what we have discussed today? Do you have any questions for me?*

*Thank you very much for your time and attention. We appreciate you sharing your thoughts and opinions with us!*

*****Is there anyone else we should be talking to? Can you provide contact details for and/or facilitate introductions with them?*****
