# Supplementary material for: Perceptions of the national school holiday programme (Holiday Activities and Food) in England as experienced by local government, service providers and users of the service: a mixed-methods study
Source: BMC Public Health. 2026 May 30;26:2425. doi: 10.1186/s12889-026-27699-1 (PMC13501641; doi:10.1186/s12889-026-27699-1)
Supplement: Supplementary file 7 [file 12889_2026_27699_MOESM6_ESM.docx]

**Additional file 6: Holiday Activities and Food Programme Interview Topic Guide – Parents/ carers**

**Consent**

If a signed consent form has not been returned by the participant, oral consent will be obtained and recorded. In this case, read the consent form to the participant and record as a separate file to the interview.

Participants will then be told about the confidentiality procedures using the following script.

*Before we get started, I’d like to tell you that our conversation today will last no more than an hour and I will be recording the conversation. The recording is to help us remember what you said. You can ask for the recording to be stopped at any point. After we have written a report about all the opinions we have heard here and with other participants, the recordings and notes will be destroyed, so none of the information that is written down and recorded can be connected to you in any way. This means that any names or phrases that you use which could identify you will be coded or anonymised in the transcripts and reports so that you can’t be identified*. *You may decline to answer any question or stop the interview at any time, and you don’t have to give a reason why.*

*Does what I have just read to you make sense? If yes proceed to*

*Are you happy for the conversation to be recorded?*

**Yes?** Proceed to interview.

**No?** Ask if they would like the information read to them again or whether there is anything they would like to be explained further. If ‘yes’ read again or offer further clarification. Then ask again:

*Are you happy for the conversation to be recorded?*

**No**: Thank participant for time and end the interview by informing them that as we are unable to record the conversation, we cannot conduct the interview.

*Thank you for taking part in the interview today. We are interested in your opinions on the Holiday Activities and Food (HAF) programme and would like to discuss these with you.*

*This interview will broadly consider the following themes:*

1) *Details of and your opinions on the programme*
2) *Attendance and engagement*
3) *Impact of the programme*
4) *Positives and negatives of the programme*

**Details of and opinions on the provision received**

1. Over the past year, when did you child attend a HAF club (e.g., summer, winter, spring, half-term?)
2. Where did it/they take place e.g., school, community centre?
3. What type/s of club were they?
4. Does your family have any additional needs (e.g., disability, SEN, language)?
a. How were these supported by the club?
5. Are you or have you attended any similar programmes that aren’t HAF?
a. Please tell us about these
b. How does the HAF programme compare to these?

**Attendance and engagement**

5) How did you hear about the programme?

prompt: When was that?

6) How did you sign up for the programme? Was that the same for each club and holiday period?
7) Do you know if the club runs during the half-term holidays? Would you attend if it did?
8) Can you please tell me your reasons for attending the HAF programme?*******

prompts:

a. the activities on offer; provided childcare; child’s friends were attending
b. what is the number 1 reason for attending? If that changed in some way, would it stop you from attending in the future?

9) Could you have attended more sessions each week and/or for more weeks of the holiday?
   a. What stopped you from attending more sessions?

Prompts: illness; transport; booking system issues; siblings?

10) Were there any days where you signed up but then were unable to attend? Why?
    a. Is there anything that could have made it easier for you to attend?
11) How can get more families to attend? And continue to attend?

**Impact of the programme**

12) What do you think you and your child would be doing if you didn’t use the programme?
13) Have you observed any effects (positive or negative) to you, your child and/or family of the programme?

Prompts:

a. Healthy eating, cooked meals
b. Physical activity – how often your child is active and type of activity
c. Fitness
d. Behaviour, confidence, self-esteem
e. Social skills, skills that will help them at school
f. knowledge and skills around buying and making healthy meals, eating as a family?
g. Attitude towards school, school attendance, educational outcomes
h. Any other outcomes (e.g., resilience, emotional skills, problem solving, attainment)?

**Positives and negatives of the programme**

14) In your opinion, what were the positives and strengths of the HAF programme?

Prompts:

a. Holiday period, timings, duration of club
b. Geographical/environmental? (e.g., location of club, access to club)
c. Social? (e.g., other children / families in attendance, reducing isolation and building relationships)
d. Club specific? (e.g., club provider, size of club, people running the club [known people i.e. community member volunteers], venue)
e. Activity specific? (e.g., activities provided, quality of activities - accessibility and appropriateness, range)
f. Opportunity e.g., getting to do something during the school holidays, trying new/different things, safe place
g. Other

15) In your opinion, what were the negatives and weaknesses of the HAF programme?

Prompts:

a. Holiday period, timings, duration of club
b. Geographical/environmental? (e.g., location of club, access to club)
c. Social? (e.g., other families in attendance)
d. Club specific? (e.g., club provider, size of club, people running the club [known people i.e. community member volunteers], venue)
e. Activity specific? (e.g., activities provided, quality of activities - accessibility and appropriateness, range)
f. Other

**Changes to the programme / best practice guidance**

16) If you were to make any changes to the programme, what would they be and why?
17) prompts:
a. Would you make any changes to when the programme runs e.g., which holiday periods (half-term)?
b. Would you want to be more involved in the programme e.g., go along to sessions to take part in activities and get fed and/or help deliver sessions?
c. Would schools be involved in the programme in any way? How?

**Closing**

*That’s all the questions I have for you today. This has been a really informative interview, thank you. Is there anything else that you think is important that we should know about regarding what we have discussed today? Do you have any questions for me?*

*Thank you very much for your time and attention. We appreciate you sharing your thoughts and opinions with us!*
