# Supplementary material for: Perceptions of the national school holiday programme (Holiday Activities and Food) in England as experienced by local government, service providers and users of the service: a mixed-methods study
Source: BMC Public Health. 2026 May 30;26:2425. doi: 10.1186/s12889-026-27699-1 (PMC13501641; doi:10.1186/s12889-026-27699-1)
Supplement: Supplementary file 8 [file 12889_2026_27699_MOESM7_ESM.docx]

**Additional file 7: Holiday Activities and Food Programme Interview Topic Guide – Children with SEND**

Thank you all for chatting with me today. I would like to ask you a few questions about the [name of HAF club] you attended.

I will be recording the chat. The recording is to help us remember what you said. You can ask for the recording to be stopped at any point. We will not share what you said with anyone else and when we write up the recording it won’t include your name.

Does what I have just said to you make sense? If yes proceed to

Are you happy for the chat to be recorded?

1. Do you like going to the [name of HAF club] during the school holidays?
   a. Is there anything you would rather be doing, instead?
   b. What makes you want to go?
   c. What makes you not want to go?
2. Have you been to any other holiday clubs?
   a. What are they like?
   b. Do you like them more or less than [name of HAF club]?
3. What were your favourite parts about the [name of HAF club]? Was your favourite thing...

Prompts:

a. The art, physical activity *(input specific type e.g., aerial, football)*
b. Food
c. Outside time
d. Being with friends
e. Staff there

4. Were there any parts of the [name of HAF club] that you didn’t like or would change next time?

Prompts:

a. The art, physical activity *(input specific type e.g., aerial, football)*
b. Food
c. Outside time
d. Being with friends
e. Staff there

5. Has anything in your life changed since you started attending the holiday club? e.g.,
a. Food you eat at [name of HAF club], food you eat at home
b. How much physical activity you do
c. How much you are able to run around / your energy (fitness)
d. Behaviour at school, how you feel about school, how often you go to school, how hard school work is
e. How you feel around other children and adults
f. How happy you feel

If you were in charge of running the [name of HAF club], you got to decide when it happens, what activities everyone does, what everyone gets to eat etc…

6. How would you do it?
7. Would you make any other changes to make the club better?

That’s all the questions I have for you today. This has been a really helpful, thank you.

Is there anything else that you think I should know about?

Do you have any questions for me?
