# Supplementary material for: Perceptions of the national school holiday programme (Holiday Activities and Food) in England as experienced by local government, service providers and users of the service: a mixed-methods study
Source: BMC Public Health. 2026 May 30;26:2425. doi: 10.1186/s12889-026-27699-1 (PMC13501641; doi:10.1186/s12889-026-27699-1)
Supplement: Supplementary file 9 [file 12889_2026_27699_MOESM8_ESM.docx]

**Additional file 8: Holiday Activities and Food Programme Focus Group Topic Guide – Children**

**Consent**

Check that signed parental consent has been received for each participant. If it has not been received for a participant, they are not able to participate.

Check that signed assent has been received for each participant. If a signed assent form has not been returned by a participant, request that the participants completed the assent form now. If the focus group is completed online, oral assent is to be obtained and recorded. In this case, read the assent form to the participant and record as a separate file to the interview.

*Thank you all for taking part in the focus group today. We are interested in what you think about the holiday club, called the HAF programme, that you recently attended.*

*I am hoping that today you will be willing to answer some questions about the HAF club including what you liked about the club and what you didn’t like about the club.*

*I would like to have a group chat about these things.*

*We will talk for around 40 minutes. A few key points before we get started:*

- *You don’t need my permission to speak but please take it in turns talking, so if someone is talking wait until they have finished to say your thoughts*
- *I would like everyone to have the chance to speak and I want to hear about different feelings: do you agree or do you feel something different? Everyone’s thoughts are interesting and important*
- *There are no right or wrong answers. Please be as honest as possible.*
- *Tell me if I don’t understand you, or if you don’t understand me*
- *You can say ‘pass’ if you don’t want to answer*

Participants will be told about the confidentiality procedures using the following script.

*I will be recording the group chat. The recording is to help us remember what you said. You can ask for the recording to be stopped at any point. If you would like the recording to be stopped or you would no longer like to take part, you can say this out loud or you can show us a thumb down* (thumb down to be shown visually at this point to participants)*.* If the focus group is online note that children can also choose to virtually show a thumb down and/or write in the chat.

NB. If a child puts a thumb down, the whole group will take a short break while the researcher speaks to the child privately about their concern. The child can then choose to re-join or opt-out of the focus group. If the child opts out, they will leave the focus group.

*All your answers are confidential, which means that they are private and will not be shared with anyone else. When we write up the recording it won’t include your name so when we look back at the conversation later we won’t know that you said your answers.*

*Does what I have just said to you make sense?* If yes proceed to

*Are you happy for the conversation to be recorded?*

**Yes?** Proceed to focus group

**No?** Ask if they would like the information again or whether there is anything they would like to be explained further. If ‘yes’ give information again or offer further clarification. Then ask again:

*Are you happy for the chat to be recorded?*

**No**: Thank participant for time and inform them that as they would not like to be recorded, they are unable to participate in the group chat.

*Ok, so I am about to start the recording.*

*Does anyone have any questions?*

I’d like to go around the group and everyone say their name, age, and one thing that they liked about the HAF club. This could be a favourite time at the holiday club or your favourite activity that you did at the club.

Thank you everyone. So I would now like to talk about the HAF club that you are attending/ have recently attended.

**Attendance and engagement**

1. Do you like going to the HAF club during the school holidays?
   a. How come?
   b. Is there anything you would rather be doing, instead of coming to the club?
   c. What makes you want to go?
   d. What makes you not want to go?
2. What do you think we could do to make more children want to go to the HAF clubs?

Prompts:

a. Location of clubs / people running the club
b. Other/more activities – which?

3. Have you been to any other holiday clubs?
   a. What are they like?
   b. How do they compare to the HAF club?
   c. Is there one that you enjoy more? Why?

**Positives and negatives of the programme**

4. What were your favourite parts about the HAF club?

Prompts:

a. Location of club, getting to the club
b. The other children who go
c. Club type / size / venue / staff
d. Activity type / range
e. Food
f. Other e.g., getting to do something during the school holidays, trying new/different things, safe place

5. Were there any parts of the club that you didn’t like or would change next time?

Prompts:

a. Location of club, getting to the club
b. The other children who go
c. Club type / size / venue / staff
d. Activity type / range
e. Food
f. Other e.g., missing out on things during the school holidays

**Impact of the programme**

6. Has anything in your life changed since you started attending the holiday club? E.g.,
a. Food you eat throughout the day, food you eat at home
b. Physical activity – how much, types
c. Fitness – how much you are able to run around / your energy
d. Behaviour at school, how you feel about school, how often you go to school, how hard school work is
e. How you feel around other children and adults
f. How happy you feel

**Changes to the programme / best practice guidance**

Imagine you were in charge of running the holiday club. You got to decide when it happens, what activities everyone does, what everyone gets to eat etc.

7. How would you do it?
8. Would you make any other changes to make the club better?

**Closing**

*That’s all the questions I have for you today. This has been a really helpful, thank you. Is there anything else that you think I should know about? Do you have any questions for me? Thank you very much for your time and attention, and for sharing your thoughts and opinions with me!*
