# Supplementary material for: Perceptions of the national school holiday programme (Holiday Activities and Food) in England as experienced by local government, service providers and users of the service: a mixed-methods study
Source: BMC Public Health. 2026 May 30;26:2425. doi: 10.1186/s12889-026-27699-1 (PMC13501641; doi:10.1186/s12889-026-27699-1)
Supplement: Supplementary file 10 [file 12889_2026_27699_MOESM9_ESM.docx]

**Additional File 9:** The Framework Method

| **Stage** | **Description** |
| --- | --- |
| 1 - Transcription | Audio recordings of interviews and focus groups were transcribed verbatim by a professional transcription company. |
| 2 - Familiarisation | Transcripts were distributed among researchers LH, LT, CKS, FD and SC, who checked the transcripts against the audio files for accuracy and gained familiarity with the data. Preliminary contextual, analytical and reflective notes were documented. |
| 3 - Coding | Initially, two Political Leads, two HAF Lead and two HAF Provider transcripts (six in total) were triple-coded; three researchers independently reviewed the transcripts, and applied a descriptive label/paraphrase (code) to sections within the transcript that were deemed relevant in relation to one or more of the research questions. Researchers met regularly to compare and discuss their coding and subsequently a preliminary list of data-driven codes was produced. Two additional transcripts from each participant group (total six) were independently coded by researchers. New codes were applied, and the preliminary list of codes was used when appropriate. Researchers met regularly to compare and refine their annotations and codes, develop a preliminary coding list, and construct a draft coding framework. This draft framework was expanded in Stage 4 to create a working analytical framework. Researchers also captured holistic impressions from the transcripts. |
| 4 - Developing a working analytical framework | After coding the first twelve transcripts, researchers met to compare and refine initial codes, and discuss the draft framework and holistic impressions. They agreed on a set of codes, categorised and defined them. The process was reflexive and iterative. Agreement between researchers led to retention of codes and categories, while disagreements were resolved through extensive discussion, with codes either kept or merged with an existing code or category. Codes were included to capture data variation. |
| 5 - Applying the analytical framework | The working analytical framework was applied to the remaining 31 transcripts, which were single-coded using existing categories and codes. Regular meetings were held to discuss, clarify, and expand the analytical framework. |
| 6 - Charting data into the framework matrix | Data was charted into a matrix; this involved creating summaries of the data by category from each transcript, identifying illustrative quotations. |
| 7 - Interpreting the data | Researchers met regularly to interpret the data, identifying characteristics and differences, discussing preliminary impressions, and mapping connections between categories and cases. An inductive and deductive approach was used to develop themes and sub-themes, which were discussed, revised, and agreed upon by all co-authors. |
